# Cortical Abnormalities and Non-Spatial Learning Deficits in a Mouse Model of CranioFrontoNasal Syndrome

**DOI:** 10.1371/journal.pone.0088325

**Published:** 2014-02-10

**Authors:** Dina N. Arvanitis, Annie Behar, Anne Drougard, Pascal Roullet, Alice Davy

**Affiliations:** 1 Centre de Biologie du Développement, CNRS UMR5547, Toulouse, France; 2 Université de Toulouse UPS, Toulouse, France; 3 Centre de Recherche Sur la Cognition Animale, CNRS, Toulouse, France; 4 Institut des Maladies Métaboliques et Cardiovasculaires (I2MC), INSERM, Toulouse, France; CNRS UMR7275, France

## Abstract

Eph receptors and their ephrin ligands play critical roles in the development of the nervous system, however, less is known about their functions in the adult brain. Here, we investigated the function of ephrinB1, an ephrinB family member that is mutated in CranioFrontoNasal Syndrome. We show that ephrinB1 deficient mice (*EfnB1^Y/−^*) demonstrate spared spatial learning and memory but exhibit exclusive impairment in non-spatial learning and memory tasks. We established that ephrinB1 does not control learning and memory through direct modulation of synaptic plasticity in adults, since it is not expressed in the adult brain. Rather we show that the cortex of *EfnB1^Y/−^* mice displayed supernumerary neurons, with a particular increase in calretinin-positive interneurons. Further, the increased neuron number in *EfnB1^Y/−^* mutants correlated with shorter dendritic arborization and decreased spine densities of cortical pyramidal neurons. Our findings indicate that ephrinB1 plays an important role in cortical maturation and that its loss has deleterious consequences on selective cognitive functions in the adult.

## Introduction

Eph receptor/ephrin signaling is a cell-cell communication pathway that plays a crucial role in embryonic development [1], yet, increasing evidence shows a role for Eph/ephrin signaling in adult physiology [2]. There are two classes of Eph receptors and ephrins (A and B) and in the adult brain, both classes have been shown to control synaptic development and plasticity in the hippocampus [3], [4], [5], [6], [7]. To date, the EphB receptors and their ligands the ephrinBs have been implicated in hippocampal LTP and in spatial learning paradigms. For example, double and triple mutant mice lacking EphB1, EphB2, and EphB3 showed significant deficits in dendritic spine formation and clustering of AMPA and NMDA receptors [8]. EphrinB3 mutant mice have impairments in hippocampal mossy fibre LTP and in hippocampal-based learning tasks [9], while ephrinB2 conditional knockout mice showed severe deficits in both LTP and in long-term depression [10], [11].

EphrinB1, which is the third member of the ephrinB family is expressed predominantly in apical progenitors in the developing cortex [12] where it has been shown to control the switch between progenitor maintenance and neuronal differentiation by regulating levels of miR-124, a pro-neuronal miRNA [13], [14]. More recently we showed that ephrinB1 is required in cortical progenitors to maintain their apical adhesion thereby ensuring the structural integrity of the developing cortex [15]. Interestingly, ephrinB1 is encoded by *EfnB1*, an X-linked gene associated with the human CranioFrontoNasal Syndrome (CFNS) [16], [17]. This syndrome is characterized by severe hypertelorism, frontonasal dysplasia, craniosynostosis and developmental delays [18], [19]. In contrast to ephrinB2 and ephrinB3, very little is known for the role of ephrinB1 in the adult brain. Previous work in cell culture has shown that ephrinB1 increases the number of dendritic spines on hippocampal rat neurons [20], regulates EphB-dependent presynaptic development on cortical rat neurons [21], and undergoes time-dependent up-regulation in lesion induced plasticity in the adult mouse hippocampus [22]. Collectively these findings suggest that ephrinB1 plays a role in synaptic development and plasticity, yet the role of ephrinB1 in cognitive function remains unknown.

Here we show that *EfnB1* deficient mice (*EfnB1^Y/−^*) display normal learning and memory in spatial learning paradigms. No deficits in locomotion or anxiety were detected in *EfnB1^Y/−^*mice; however, they exhibited a specific impairment in non-spatial learning and memory tasks. Given that we observed no impairment in spatial learning, which strongly implicates the hippocampus (reviewed in [23], [24]) and rather observed learning impairments that are strongly associated with cortical functioning [25], [26], [27], [28], emphasis was placed on analyzing the cortex, specifically the perirhinal cortex, in these mutants. Surprisingly, we established that ephrinB1 does not control learning and memory through modulation of synaptic plasticity in adults, since it is not expressed in the adult brain. Further scrutiny of the adult cortex of *EfnB1^Y/−^* mice showed supernumerary neurons, with increased interneuron number, and decreased dendritic complexity in cortical pyramidal neurons as compared to wild-type (*EfnB1^Y/+^*) littermates. Altogether, our findings indicate that the absence of ephrinB1 results in increased neuron numbers with changes in dendritic morphology in the postnatal cortex that correlate with a selective learning and memory deficit.

## Materials and Methods

### Animals

Wild-type (WT : *EfnB1^Y/+^*), and mutant *EfnB1^Y/−^* male mice where generated as described [29] and kept in a mixed 129S4/C57BL/6J genetic background Heterozygote females were crossed with *EfnB1^Y/+^* or *EfnB1^Y/−^* males to generate all genotypes. All animal procedures were approved by the Midi-Pyrénées Animal Experimentation Ethics Committee (MP/07/21/04/11).

### Behavioural Testing

All experiments were performed during the light phase. Three- to 4-month old mice (n = 12 per genotype) were familiarized to the experimenter and mice were subject to two behavioral tasks; one non-stressing test (object recognition or object location) and one aversive task (Morris water maze, passive avoidance or fear conditioning). The order of experiments was pseudo randomized to avoid any influence of test order. Locomotor activity was analyzed during the 10 min familiarization phase of the object recognition or object location tasks. Anxiety was evaluated based on percent time spent in the open arms of the elevated plus maze.

#### Morris water maze

Spatial memory testing was conducted as described [30], [31]. Briefly, mice were introduced to a circular pool (110 cm in diameter) filled with water made opaque. Subjects were trained to locate the hidden platform, which was submerged 0.5 cm below the water. One mass-training procedure was performed to unambiguously separate acquisition from consolidation processes. With this massed procedure, we have the same hippocampal involvement during acquisition and consolidation as found in the distributed procedure [32], [33]. The procedure included one training session composed of 4 blocks, each consisting of three consecutive trials. The phase between consecutive blocks was 15 to 20 min long, during which the mouse was returned to its home cage. Twenty-four hours post acquisition, memory was assessed during a single one minute probe test in the absence of the platform.

#### Object recognition

The procedure consisted of three different phases as described [34]. Briefly, a familiarization phase in which each mouse was placed in the empty square open-field for 10 min. A sample phase, 24 h later, in which two identical metallic objects were placed in the middle of the open-field. A test phase, 24 h later whereby mice were reintroduced into the arena and exposed to two objects, a familiar object and a novel object, to test recognition memory. The percent time spent exploring the novel object was calculated as a preference index to measure novel object recognition Performance in this non-spatial task, in contrast to the object location task, is not affected by hippocampal lesions [35], [36] unless conducted in a complex spatial environment [36]. To avoid this bias, we used the original procedure described by Ennaceur and Delacour [35], which we have previously demonstrated does not require an intact hippocampus [37].

#### Object location

The same open-field with the same environment was used as that of the object recognition task. A similar procedure was employed except that one of the two identical objects was moved to a novel location [34]. The percent time spent exploring the displaced object was calculated as a preference index to measure spatial memory.

#### Fear conditioning

Mice were placed in the fear-conditioning apparatus chamber for 5 min and 30 sec. After a 2-min exploration period, a sound (CS) was emitted for 30 sec and a foot-shock of 0.7 mA (US) was superposed to the tone during the last 2 sec. After 2 min, the paired CS-US was repeated. Twenty four hours after conditioning, context-dependent freezing rate was measured in the conditioning chamber for 4 min. Three hours later, context-independent (tone dependent) freezing rate was measured. Specifically, mice were placed in a modified context; 2 min after their introduction in the modified chamber, mice received a 2-min tone presentation. Freezing rates to CS or US were calculated for *EfnB1^Y/+^* and *EfnB1^Y/−^* mice as described [38].

#### Passive avoidance

The apparatus consisted of a rectangular box divided into an illuminated safe compartment and a dark shock compartment. Subjects were individually placed into the illuminated compartment and when subjects entered completely inside the dark box, a foot shock (280 µA for 2 sec) was administered. Subsequently, the mouse was placed in its home cage. Retention was measured 24 h later by placing the mouse in the light compartment and measuring the latency to enter the dark box.

### Brain Sample Preparations

Four to five month old *EfnB1^Y/+^* and *EfnB1^Y/−^* mice month old mice, following behavioural testing, were anesthetized with an injection cocktail of 3∶3∶1 ketamine (100 mg/ml)/xylazine (20 mg/ml)/acepromazine (10 mg/ml) at a dose of 0.01 ml injection cocktail/g body weight and intra-cardially perfused with 0.9% saline followed by 4% formaldehyde in phosphate buffered saline (PBS). The dissected brains post-fixed in 4% PFA at 4°C. All sample brains were removed from PFA and either equilibrated in 70% ethanol and embedded in paraffin or equilibrated in sucrose and embedded in OCT blocks for cryostat sectioning. Alternatively, *EfnB1^Y/+^* and *EfnB1^Y/−^* mice, at specified ages were sacrificed by cervical dislocation and quickly weighed. The brains were removed, weighed and harvested in RNA Later® (Ambion) until use. Coronal sections (7 µM) were obtained and placed on Superfrost microscope slides (Fisher Scientific) and stored at room temperature until use.

### Cresyl Violet Staining

Multiple serial sections were Nissl-stained to provide a qualitative view of the cortical and hippocampal structures. The slides were immersed in 25°C cresyl violet for 10 min, washed vigorously in rapid exchanges of distilled water to remove the excess cresyl violet, dehydrated in graded ethanol, cleared in xylenes, and coverslipped. Images were acquired using a Nikon Eclipse 80i.

### Immunohistochemistry

Sections used for immunohistochemistry were blocked in 3% goat serum in PBS. Primary antibodies were against ephrin-B1 (1∶50, R&D Systems), NPY (1∶5000, Sigma Aldrich), PV (1∶5000, Swant), Calretinin (1∶5000, Swant), NeuN (1∶600, Millipore). Primary antibodies were visualized by secondary antibodies conjugated with Alexa (1∶300, Jackson Immunoresearch), followed by nuclear counterstaining with Draq5 (Vector Labs) for quantification of total cell number. Images were acquired using a Confocal LEICA SP2 or LEICA SP5.

### Neuronal Cell Counts

The mouse brain atlas was used to identify different regions of the cortex, hippocampus, amydgala and striatum. Serial coronal sections (7 µm) were obtained. NeuN staining was performed on every 20^th^ µm -section from postnatal and adult brains (n = 3/genotype/age). For quantification of cells, images were taken with a 40× objective using a confocal microscope (Leica SP2 or SP5). Cell counts included all fully visible cells within the 100 µm^2^ counting frame using the cell counter plugin for ImageJ. For each cortical area the number of NeuN+ cells was quantified in 3-nonoverlapping 100 µm^2^ counting boxes starting at the pial surface. Emphasis was placed on sections containing perihinal cortex. For the hippocampal CA1 and CA3 a 100 µm^2^ counting box was placed over each region and NeuN-positive cells were counted manually using the cell counter plug-in from ImageJ. For the amygda two non-overlapping 100 µm^2^ counting boxes were placed in the amygdala region at the level of 2.34 mm to 1.62 mm posterior to the interaural plane. For the striatum, four coronal sections at the level of 3.94 mm posterior to the interaural plane were analyzed. Three non-overlapping 100 µm^2^ counting frames were placed vertically starting at the corpus callosum. Interneuron staining was performed on every 10^th^ section between the interaural planes 3.10 mm to 1.10 mm (n = 6/genotype). A 100 µm^2^ counting box was placed across three different counting regions of interest in the frontal, somatosensory or perirhinal cortices. Triplicate cell counts were performed in each region. Furthermore, the cortical thickness was measured as the shortest distance between the grey/white matter boundary and pial surface. Triplicate measurements were performed on 10 sections per sample; n = 3 per genotype.

### Golgi Cox Impregnation

Brain tissues were removed and processed by Golgi-Cox staining, using procedures described previously [39]. Briefly, brains were first stored in the dark for 14 days in Golgi-Cox solution followed by 3 days in 30% sucrose. The brains were sectioned 200-mm thick on the coronal plane using a vibratome. Sections were collected on cleaned, gelatin-coated slides and stained in ammonium hydroxide for 30 min, followed by Kodak Fix for film for another 30 min and finally were washed with water, dehydrated, cleared and mounted using Permount (Fischer Scientific). Slides were coverslipped and allowed to dry before quantitative analysis. Several pyramidal neurons impregnated with the Golgi solution were readily identified in the cortex by their characteristic triangular soma shape and numerous dendritic spines. Four to five neurons per animal were reconstructed by ImageJ software. For spine quantification, a 100× oil-immersion objective was used to identify spines in dendrites longer than 10 µm. Quantification of the number of primary dendrites (defined as dendrites longer than 21 µm emanating directly from the soma) and total dendrites (defined as the amount of all dendritic branches) was done on images acquired with 25× objective, in which a circle was drawn around the cell body and the number of dendrites crossing each circle (primary dendrites) and the total branches were manually counted. Quantitative analysis for spines was performed using NeuronStudio and ImageJ in images acquired with 100× objective. The dendritic branching patterns were analyzed using Sholl’s method of regularly spaced concentric circles centred on the neuronal soma [40]. The number of dendritic intersections crossing each 20 mm-radius circle progressively more distal from the soma was counted. For each of these parameters, an average was calculated for each animal. Means for the *EfnB1^Y/+^* and *EfnB1^Y/−^* groups were then obtained from these individual values. Spine densities were calculated as mean numbers of spines per micrometer per dendrite per neuron in individual mice per group.

### Quantitative RT-PCR

RNA from embryonic and adult cortices was harvested in RNA Later® (Ambion) and total RNA was extracted using Trizol® (Invitrogen) according to the manufacturer’s instructions. Messenger RNA expression was measured by qRT-PCR using a Bio-Rad thermo-cycler. Reverse transcription (RT) was perfomed using Superscript® III (Invitrogen) with 1 µg of total RNA per reaction. For the qRT-PCR reaction the resultant cDNA was diluted 1∶1000. Each RT step was performed in duplicate and the qRT-PCR in triplicate for each RT reaction. RT-PCR was performed using SYBR Green JumpStartTM (Sigma-Aldrich). Relative values were calculated by the 2-ΔdCT method. Values were normalized to β-Actin or GAPDH mRNA levels. Results in adult tissues are standardized to *EfnB1* expression values at E16.5. Primer sequences were as follows:

*EfnB*1F:TGAAGGTTGGGCAAGATCC, R:GGTTCACAGTCTCATGCTTGC;

*EfnB*1F:GACAAGCTGGACATCATCTGC, R:GCCGCCCGCTGCTGTGT;

*EfnB*1F:TTGGCCAAGAACCTGGAG, R:GCCCTTCCCACTTAGGAACT.

*Actin*F:TCGACAACGGCTCCGGCAT, R:AAGGTGTGGTGCCAGATTTTC.

*Gapdh*F:TGAAGGTCGGTGTGAACGGATTTGGC, R:CATGTAGGCCATGAGGTCCACCAC.

Note that different primer sequences for *EfnB1* were used to confirm expression levels in adult cortices.

### Statistical Analysis

Mean, standard deviation and p values were calculated using Excel software. For behavioural analyses SYSTAT 11.0 statistical software was used. The results were analyzed using one or two-way ANOVAs, or a repeated measure ANOVA when appropriate. *Post-hoc* multiple comparisons were carried out when allowed, using Tukey’s Honestly Significant Distance (HSD) test. Student t-test was performed to determine significant differences between samples. A value of p<0.05 was considered statistically significant. For dendritic complexity and spine density, the results are presented as mean ± S.E.M. Statistical differences were determined by Student’s t test for two-group comparisons.

## Results

### Spatial Memory is Unaffected in *EfnB1* Mutant Mice

**Figure 1:**
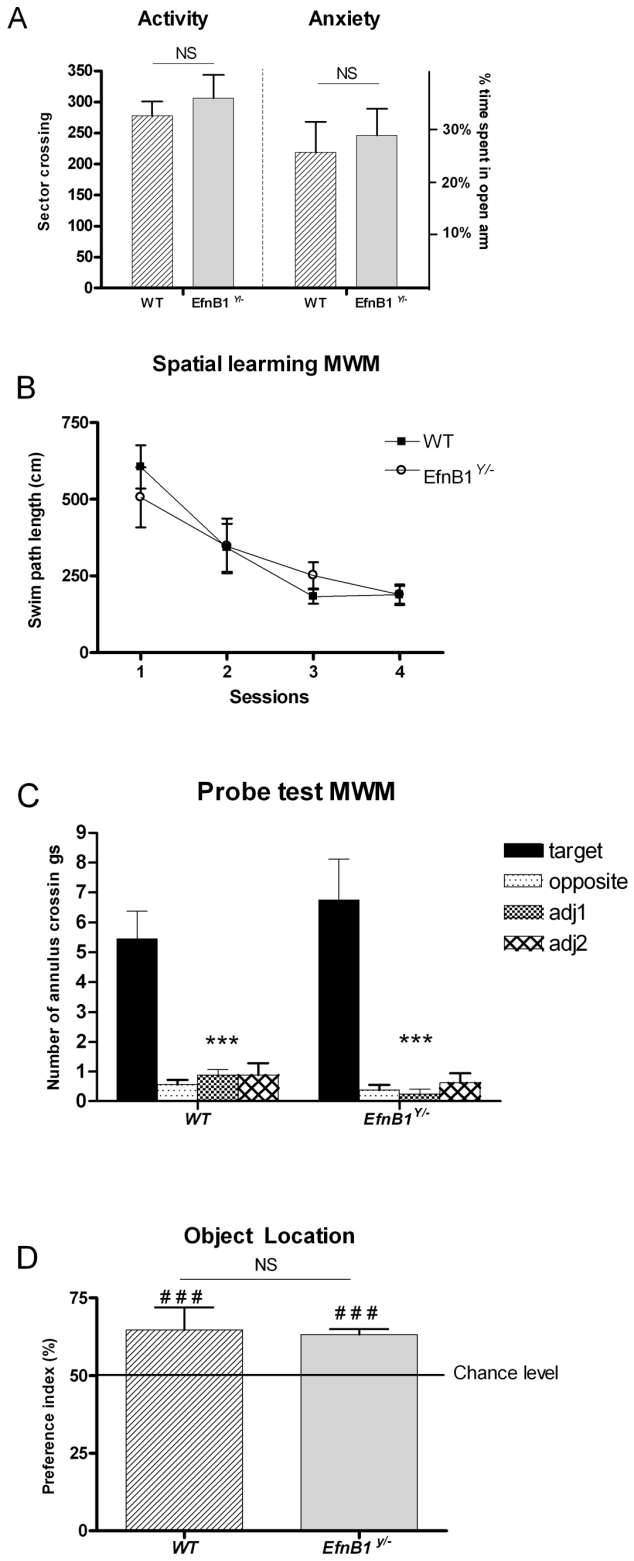
General behaviour and spatial memory are preserved in *EfnB1* mutant mice. *A.* Locomotor activity and anxiety levels in *EfnB1* mutant (*EfnB1^Y/−^*) mice as compared to control (WT) mice. *B.* Graph presenting swim path length as a function of individual training sessions (S1–S4). Throughout the training sessions, *EfnB1^Y/−^* mice learned equally well to locate the hidden platform and exhibited decreasing swim distances over blocks of trials (F_3,45_ = 12.58, *p*<0.001). *C.* Number of annulus crossings during probe tests. All groups of mice showed similar preference for the target zone where the platform was located during training sessions as compared to the adjacent (Adj1 and Adj2) and opposite zones (target *vs* others, **p<0.01; ***p<0.001). *D.* Performances in the object location task are expressed as the group mean (± SEM) preference index. The horizontal line represents equal exploration of the two objects. *EfnB1^Y/−^* and WT mice presented the same level of reaction to the object displacement (F_1,17_ = 0.139, *p* = 0.714) and spent significantly more time exploring the displaced object than the non-displaced one (^###^p<0.001; index *vs* chance level (50%)). NS: not significant.

The general behaviour of *EfnB1^Y/−^* mice is comparable to that of *EfnB1^Y/+^* mice, as we observed no significant differences in the level of locomotor activity or anxiety between *EfnB1^Y/−^* mice and control mice (Fig. 1*A*). Spatial learning and memory competences of *EfnB1^Y/−^*males were assessed using the Morris Water Maze (MWM). *EfnB1^Y/+^* and *EfnB1^Y/−^* mice performed equally during the acquisition phase (Fig. 1*B*) and during the probe test 24 h after acquisition (Fig. 1*C*). These findings demonstrate that *EfnB1^Y/−^*mice showed no deficits in spatial learning and memory as determined by the MWM.

To validate the notion that loss of ephrinB1 does not influence spatial memory, *EfnB1^Y/+^* and *EfnB1^Y/−^* male mice were further assessed in the object location task. During the retention phase, both *EfnB1^Y/+^* and *EfnB1^Y/−^* mice spent significantly more time exploring the displaced object, showing a preference index significantly different from chance level (50%; Fig. 1*D*). Therefore, our findings indicate that spatial learning and memory, which are hippocampus-dependent, were normal in absence of ephrinB1.

### Non-spatial Memory Deficits in *EfnB1* Mutant Mice

**Figure 2:**
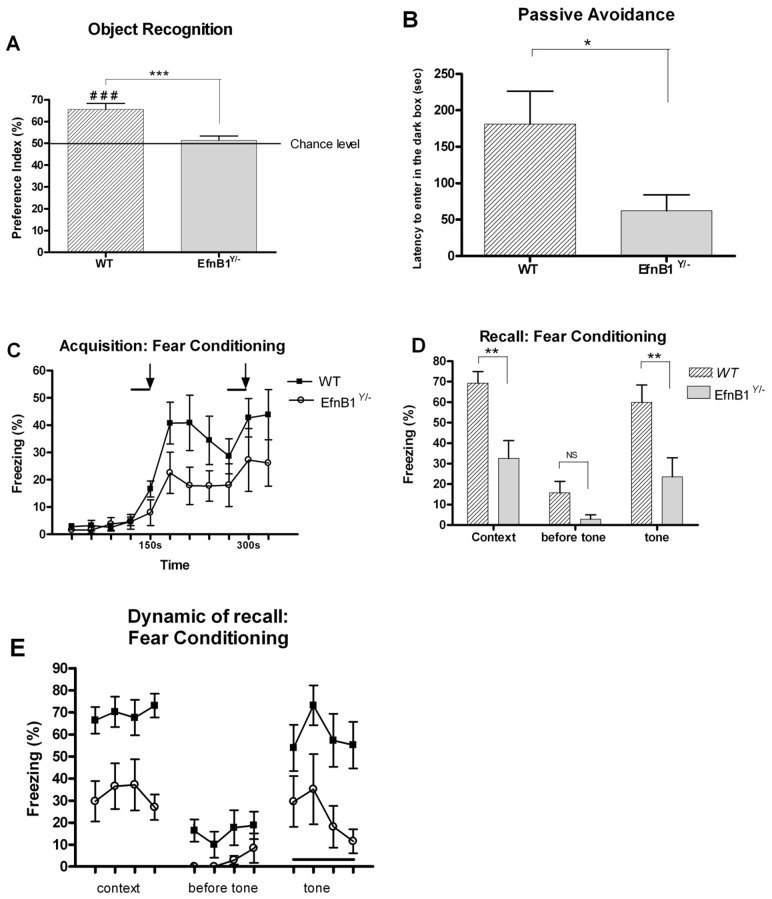
*EfnB1* mutant mice present a deficit in non-spatial memory. *A.* Performances in the object recognition task are expressed as the group mean (± SEM) preference index. The horizontal line represents equal exploration of the two objects. *EfnB1* ^Y/−^male mice exhibited severe deficits in this task (F_1,14_ = 17.890 p<0.001) and were not able to distinguish the new object. *B.* In the passive avoidance paradigm, *EfnB1^Y/−^* male mice exhibit decreased memory performance relative to wild-type littermates (F_1,15_; 6.002 p = 0.027). *C.* Acquisition curve for the percentage freezing rate in the fear conditioning task. *EfnB1^Y/−^* mice in the 2 groups showed similar levels of freezing before the presentation of any tones (F_3,45_ = 0.272 p = 0.845). During the two tone presentation, the freezing performances are not different between the two groups (first tone: (F_1,15_ = 2.282 p = 0.110;second tone: F_1,15_ = 1.472 p = 0.244) and subsequent to the first tone presentation, all mice showed increased freezing (F_6.90_ = 3.781 p = 0.002). Even though lower levels of freezing behaviors were observed in *EfnB1* ^Y/−^ mice, this difference was not significant (F_1,15_ = 3.48 p = 0.082). Solid lines represent tone and arrows indicate the presentation of a foot shock *D.* 24 h following conditioning, male mutant mice were impaired in contextual fear conditioning (F_1,15_ = 13.694 p = 0.002) and in cued fear conditioning (F_1,15_ = 7.921 p = 0.013). E During the two tests, the dynamic of the freezing response is not different between the two groups indicating that the deficit in performances reflects more a global decrease in the quantity of freezing than a deficit in delayed recall (contextual: F_3,45_ = 1.268 p = 0.297; cued: F_3,45_ = 0.636 p = 0.596). ^**^ *P*<0.01. NS: not significant.

We next studied non-spatial learning and memory competencies in *EfnB1^Y/−^*mice. In the object recognition test, mutant and control males spent an equal amount of time exploring the two identical objects during the training phase (data not shown). During the retention test *EfnB1^Y/+^* mice spent significantly more time exploring the novel object as opposed to the familiar one, so that the preference index was significantly different from chance level (50%). In contrast, the preference index for *EfnB1^Y/−^* mice was not different from chance level indicating that these animals were not able to recognize the novel object (Fig. 2*A*). These results suggest that *EfnB1^Y/−^* mice exhibited a non-spatial memory deficit. To further explore non-spatial learning and memory in *EfnB1* mutants, the subjects were examined in the passive avoidance and fear conditioning paradigms. In the passive avoidance paradigm, *EfnB1^Y/−^* mice exhibited shorter latencies to enter the dark chamber during the retention test than did the corresponding *EfnB1^Y/+^* controls (Fig. 2*B*) therefore indicating that mutant males displayed a significant memory retention deficit in this aversive task. We further tested these mice in the fear conditioning paradigm. Interestingly, we found that *EfnB1^Y/−^*mice demonstrate impairments both in contextual and cued conditioning tests (Fig. 2*C, D*), thus the deficit is not specific to hippocampus-dependent (context) memories [*41*]. Altogether these findings indicate that loss of ephrinB1 leads to impairments in non-spatial learning and memory that preferentially implicate the cortex.

### Behavioural Deficits Indirectly Correlate with EphrinB1 Function

**Figure 3:**
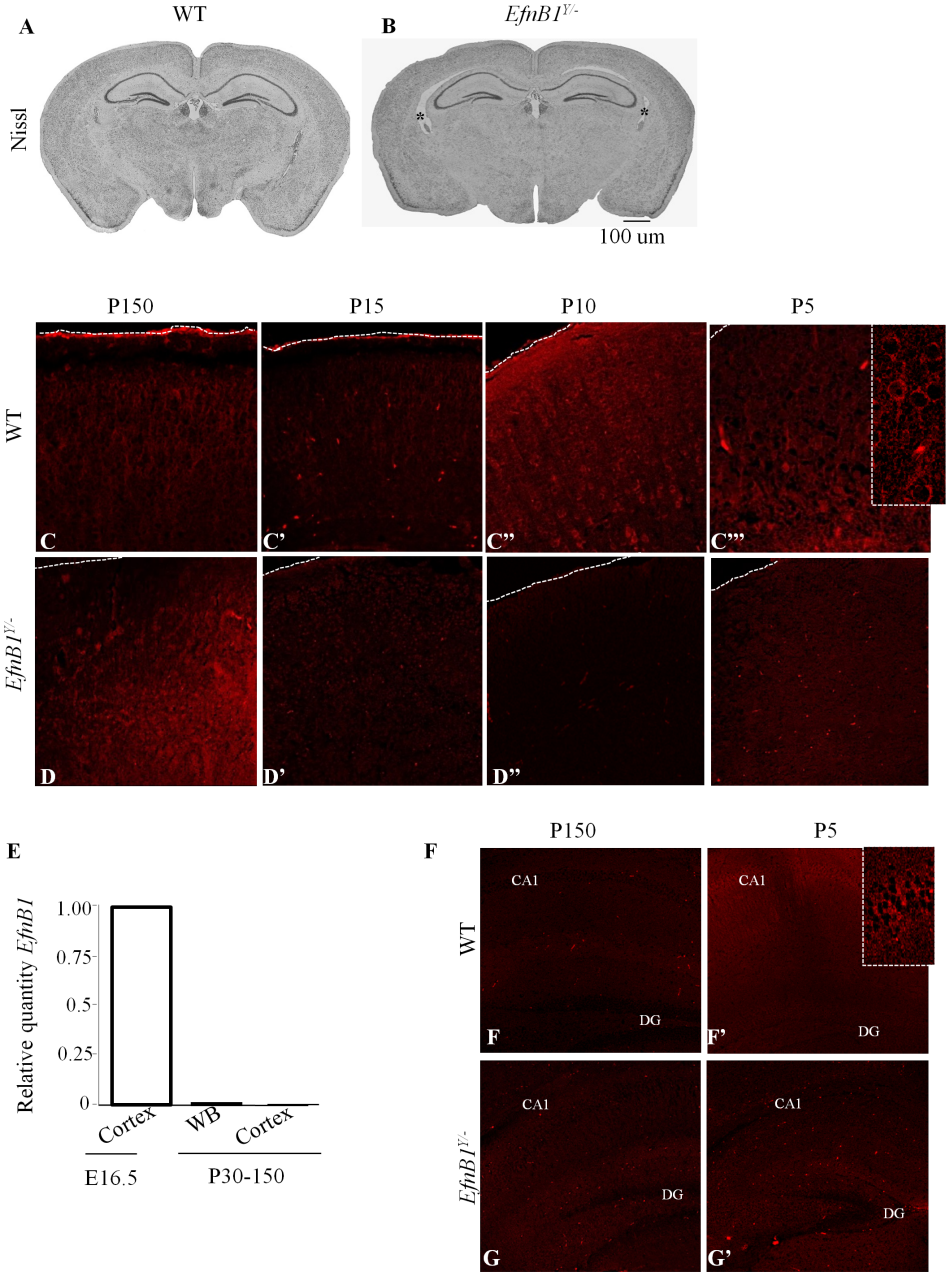
EphrinB1 is not expressed in the adult cortex. *A, B.* Representative coronal section of the brain from wild type (*A*) and *EfnB1* mutant (*B*) adult mice showing normal gross morphology of the brain. Both mutant and wild type brains have similar sizes. A slight enlargement of the ventricles was observed in some of the mutant brains (*B*, asterisks). *C–D’’’.* Immunohistochemistry for EphrinB1 at noted postnatal (P) dates in *EfnB1^Y/+^* and *EfnB1^Y/−^* cortices. EphrinB1 is not detected in *EfnB1^Y/+^* adult cortices (*C*), rather strong, positive signals are observed at P5 (*C”’*) and decline by P15 (*C’*) in *EfnB1^Y/+^* samples. *E*. Quantitative RT-PCR of P30–150 whole brain (WB) extracts and cortical extracts were analysed for *EfnB1* expression and compared to control embryonic (E) day 16.5 cortical extracts that express *EfnB1.* n = 3/age. *F–G’.* Immunohistochemistry for ephrinB1 in *EfnB1^Y/+^* and *EfnB1^Y/−^* hippocampi at P5 and P150. EphrinB1 is not detected in *EfnB1^Y/+^* adult hippocampi (*F*), but observed in the CA1 region at P5 (F’; inlet).

To establish the neuroanatomical basis of the observed behavioural deficits in *EfnB1* mutants, we performed a histological analysis of adult brains from *EfnB1^Y/+^* and *EfnB1^Y/−^* mice. *EfnB1* mutants displayed similar brain:body mass indices compared to littermate controls (*EfnB1^Y/+^*: 0.211±0.05, *EfnB1^Y/−^*0.208±0.07). The gross morphology of *EfnB1^Y/−^*brains was normal (Fig. 3*A, B*); however, 28% (4/14) mutants demonstrated a slight enlargement of the ventricles (Fig. 3*B*). We did not observe agenesis of the corpus callosum in *EfnB1^Y/−^*mice, even though it has been previously reported by others [*42*]. This discrepancy is likely due to the different genetic backgrounds of the mice under study.

The *EfnB1^Y/−^* mice displayed no deficit in any of the spatial learning paradigms that are known to strongly involve the hippocampus [23], [24]; but rather showed impairment in non-spatial learning tasks that have been shown to be largely independent of hippocampal involvement but involve the cortex [35], [36]. To better understand the putative role of ephrinB1 in non-spatial learning and memory, we performed immunohistochemical staining to establish the expression pattern of ephrinB1 in the adult brain. Surprisingly, we could not detect ephrinB1 in the adult WT cortex or in the adult WT hippocampus at the stage where behavioural studies were performed (Fig. 3). These findings indicate that the learning and memory deficit observed may be independent of ephrinB1 expression in the adult brain. To further assess the spatio-temporal expression pattern of ephrinB1 in the postnatal brain we performed qRT-PCR and immunohistochemical analyses and found that while ephrinB1 is expressed at P5 in the cortex, its levels decrease by P10 and it is no longer detected in the P15–P150 brain (Fig. 3*E*). Similar to our findings for the cortex, we found that ephrinB1 is expressed throughout the hippocampus in the early postnatal brain and not expressed in the adult (Fig. 3*F, G* *).* Therefore, ephrinB1 expression coincides with early postnatal brain maturation.

### Supernumerary Neurons in the Cortex of Adult *EfnB1^Y/−^* Mutant

**Figure 4:**
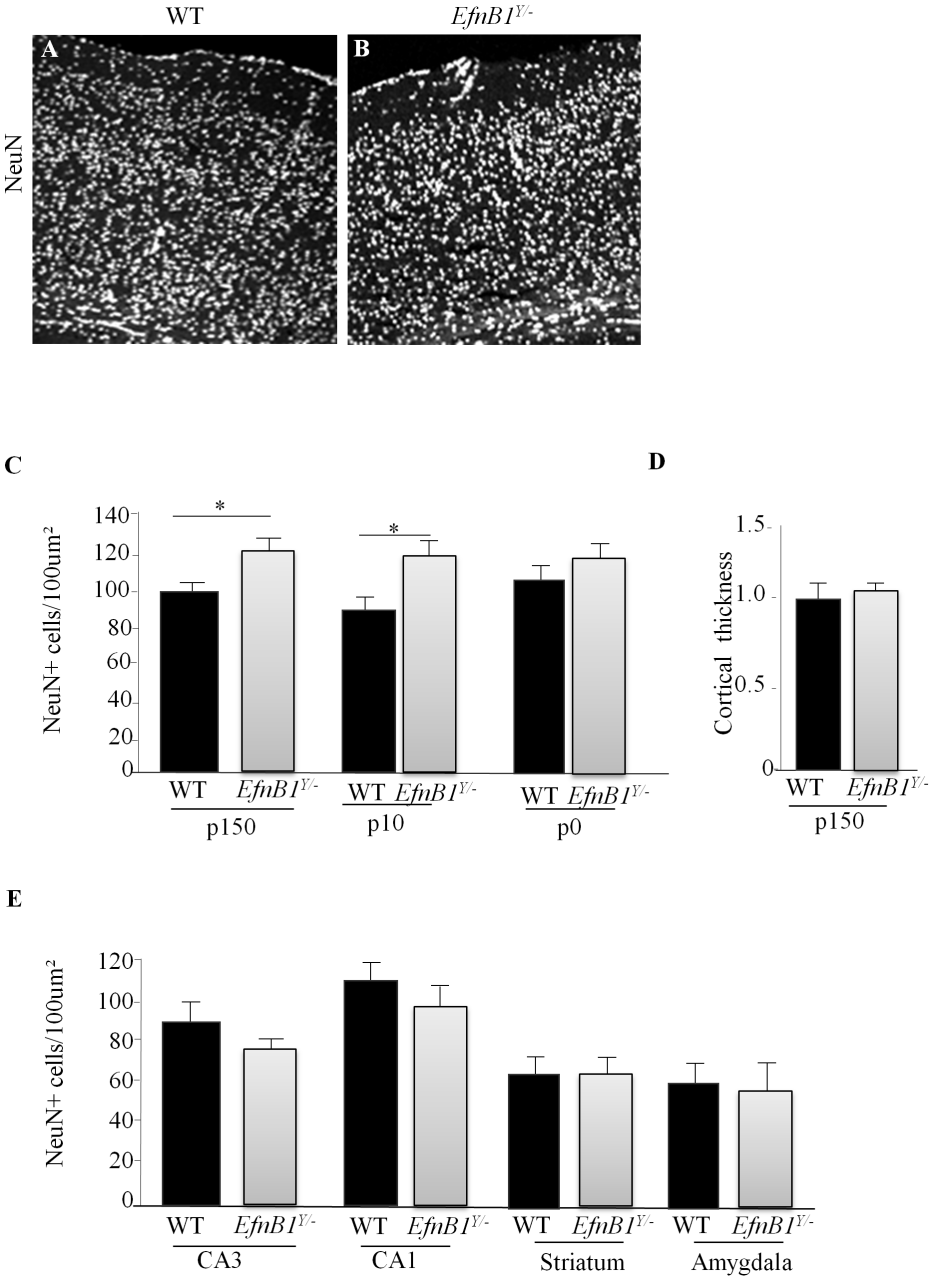
Changes in cell number in *EfnB1* mutant cortices. *A, B.* Representative images of cortical sections from *EfnB1^Y/+^* (WT; *A*) and *EfnB1^Y/−^* (*B*) mice at P150 stained for NeuN. Shown are areas of the frontal cortex. *C.* Quantification of NeuN stained cells demonstrating an increase in NeuN-positive cells in P150 *EfnB1^Y/−^* mice, P10 *EfnB1^Y/−^* mice and not at P0 stages. * p<0.05. *D.* Ratio of cortical thickness in *EfnB1^Y^* ^***/−***^ mice as compared to controls. *E.* Quantification of NeuN positive cells in the CA3 and CA1 region of the hippocampus, and in the striatum and amygdala in P150 *EfnB1^Y/+^* and *EfnB1^Y/−^* mice.

Given that ephrinB1 is not expressed in the adult brain we asked whether the cortex exhibited anomalies at the cellular level that may correlate with the non-spatial learning and memory deficit. We performed immunohistochemistry with the pan-neuronal marker (NeuN) at various postnatal stages and observed that starting at P10, a significantly greater number of NeuN positive cells were present in the cortex of *EfnB1^Y/−^* compared to *EfnB1^Y/+^* mice (Fig. 4*A–C*). This difference in neuron number was still present at p150, where counts were conducted in all cortical regions; the frontal cortex, the somatosensory 1 cortex (S1), the somatosensory 2 cortex (S2), and the perirhinal cortex (Phr). Importantly, this increase in cell number did not correlate with an increased thickness of the mutant cortex (Fig. 4*D*). To determine whether the increase in neuron number was specific to the cortex, we quantified NeuN positive cells in the striatum, amygdala and hippocampal CA1 and CA3 regions. We observed no statistical difference in NeuN positive cells in any of these structures in *EfnB1^Y/−^* compared to *EfnB1^Y/+^* mice (Fig. 4*E*). Altogether, these results indicate that loss of ephrinB1 leads to a postnatal increase in cortical neuron number.

### Increased Interneuron Number in Adult *EfnB1^Y/−^* Mutants

**Figure 5:**
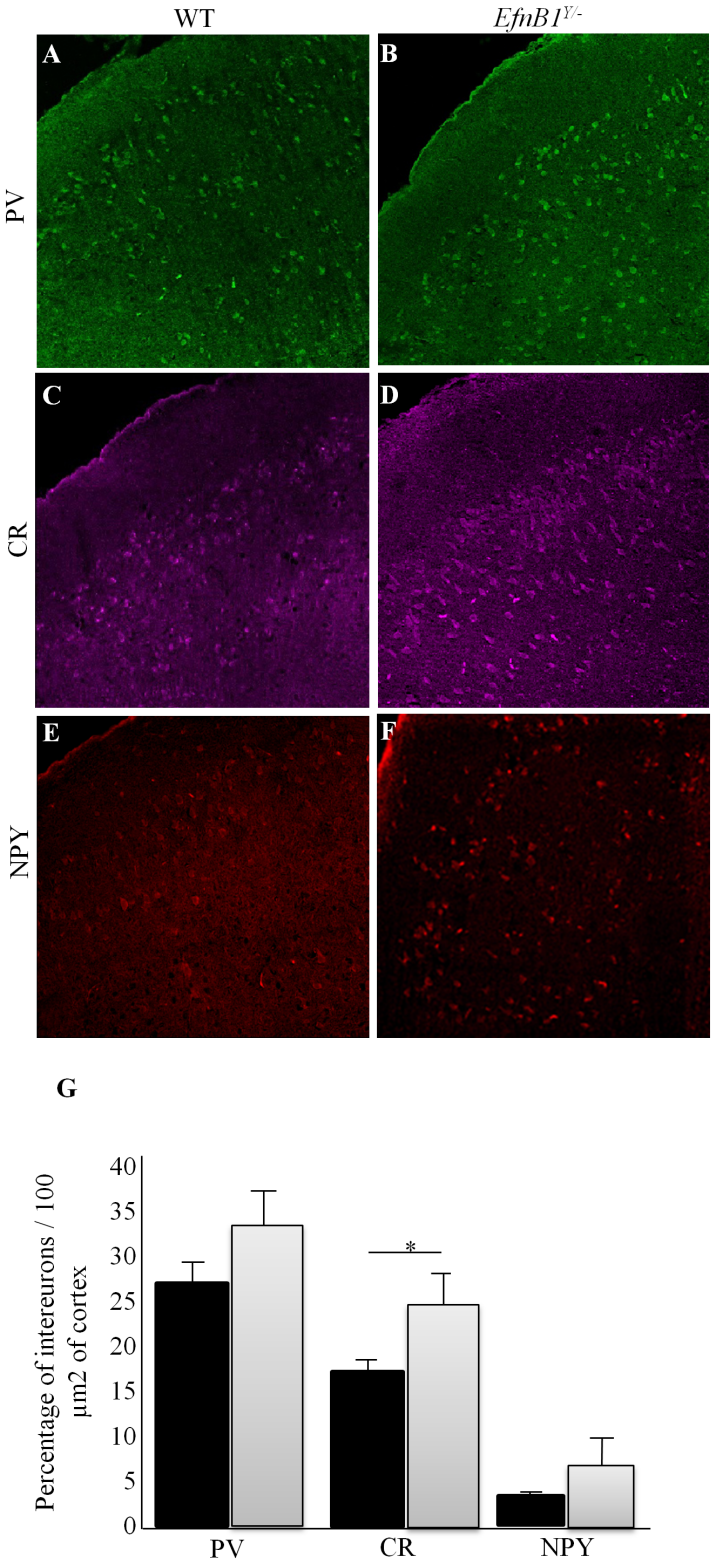
Increased interneuron number in adult *EfnB1^Y/−^* mutants. *A–F.* Representative immunofluorescent images of *EfnB1^Y/+^* (*A, C, E*) and *EfnB1^Y/−^* (*B, D, F*) mutant somatosensory S2 cortices stained for PV (*A, B*), CR (*C, D*) and NPY (*E, F*) The percent of interneuron numbers per 100 micro(µ)m^2^ is shown for the S2 region of *EfnB1^Y/+^* versus *EfnB1^Y/−^* samples. *G.* We observed an overall increase in PV+, CR+ and NPY+ neurons in *EfnB1^Y/−^* mutants as compared to *EfnB1^Y/+^* in all cortical regions studied. A significant increase was detected for CR+ neurons in the *EfnB1^Y/−^* S2 cortical region. * p<0.05.

Changes in interneuron numbers have been widely implicated in a number of cognitive deficits [43], therefore we quantified cortical interneuron populations including parvalbumin (PV+; Fig. 5*A, B*), calretinin (CR+; Fig. 5*C, D*), and neuropeptide Y (NPY+; Fig. 5*E, F*) interneurons in *EfnB1^Y/+^* and *EfnB1^Y/−^* mutants. Counts were conducted in all cortical regions; the frontal cortex, S1, S2, and Phr of *EfnB1^Y/+^* and *EfnB1^Y/−^* mice. Importantly, only the proportion of CR+ neurons, specifically in the S2 cortical region, was significantly increased in absence of ephrinB1 (Fig. 5*G*).

### Decreased Dendritic Arborisation and Synaptic Density in *EfnB1* Mutants

**Figure 6:**
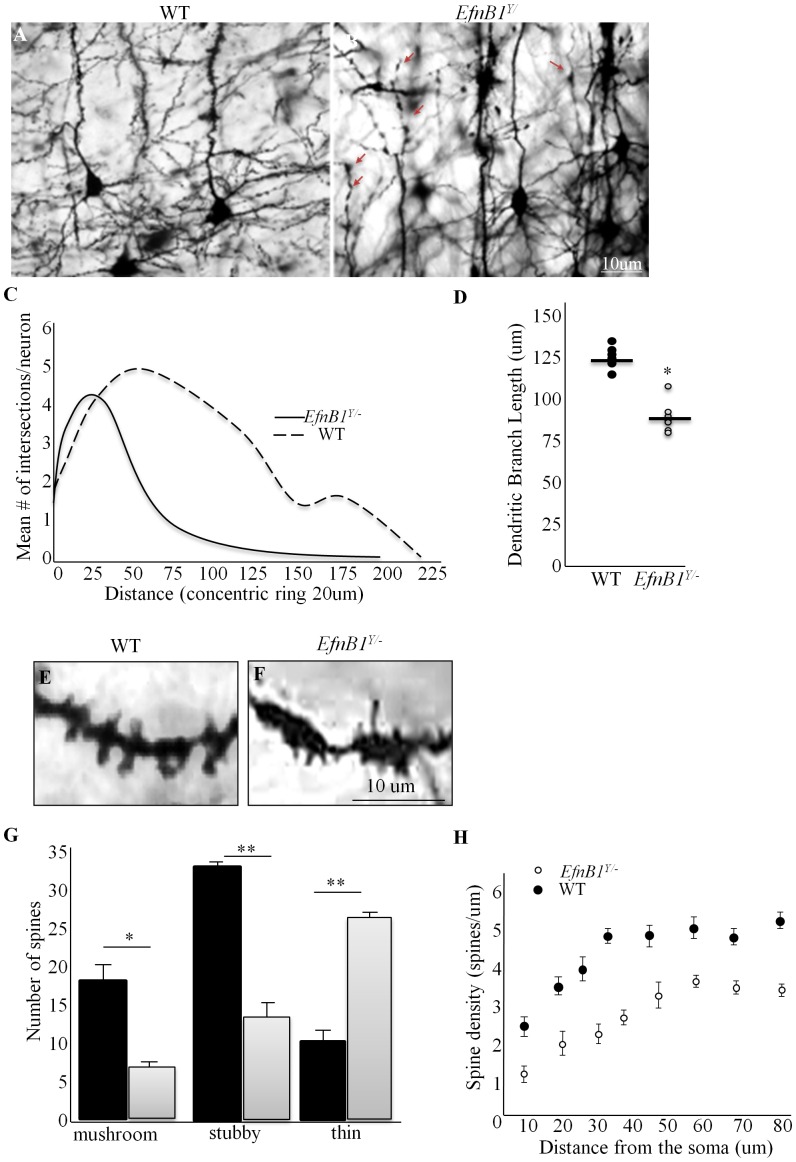
Comprised dendritic arbors in ephrinB1 mutant cortical pyramidal cells. *A, B.* Representative photomicrographs of golgi-cox impregnated S2 pyramidal neurons from *EfnB1^Y/+^* (*A*) and *EfnB1^Y/−^* (*B*) cortices. Note the presence of dendritic varicosities on dendrites from *EfnB1^Y/−^* samples (arrows). *C.* Sholl analysis of the structure of dendritic arbors. Mean number of dendritic intersections of layer II/III cortical pyramidal neurons show a significant decrease in the number of intersections in pyramidal neurons from *EfnB1^Y/−^* cortex at longer distances from the cell soma. Note that majority of dendritic intersections in *EfnB1^Y/−^* cortex were found proximal to the soma, at a distance of ∼20–50 µm compared to *EfnB1^Y/+^* cortex. *D.* Quantitative analysis of dendritic branch length shows a statistically significant decrease in the branch length of pyramidal neurons from *EfnB1^Y/−^* samples compared to controls (*EfnB1^Y/+^* = 125 µm±7; *EfnB1^Y/−^* = 88 µm±12; p<0.01). *E, F.* Higher magnified photomicrographs of *EfnB1^Y/+^* (*E*) and *EfnB1^Y/−^* (*F*) pyramidal neurons in the S2 field. Note the varicosities in dendrites from *EfnB1^Y/−^* samples. *G.* Quantitative analysis of the number of morphologically different spines on the basilar tree of cortical layer II/III pyramidal neurons in *EfnB1^Y/+^* and *EfnB1^Y/−^* samples. A significantly higher number of thin spines are observed on neurons from *EfnB1^Y/−^* mice. *H.* Analysis of the total density of spines along a dendrite showed fewer spines throughout the entire length of the basal dendrites. Each dot represents 4–5 neurons per individual. n = 5/genotype. * p<0.05, **p<0.001.

We next sought to determine whether changes in neuronal number correlated with modification of synaptic contacts in the cortex of *EfnB1^Y/−^*mice. The dendritic complexity of the layer II/III pyramidal neurons from three different regions of the mouse cortex: the frontal cortex, S1, S2, and Phr cortical neurons was analysed in adult *EfnB1^Y/+^* and *EfnB1^Y/−^* mice (Fig. 6*A, B* *)*. Sholl analysis showed a significant difference in the number of intersections at longer distances from the cell soma (Fig. 6*C*). Further, the dendritic branching of neurons from *EfnB1^Y/−^* mice was less complex than that observed in *EfnB1^Y/+^* animals. Moreover, the majority of dendritic intersections in the cortex of *EfnB1^Y/−^* mice were found proximal to the soma, at a distance of ∼20–50 µm compared to *EfnB1^Y/+^* (Fig. 6*C*). Quantitative morphological analysis of neurons in the cortex of *EfnB1^Y/+^* and *EfnB1^Y/−^* mice showed that the length of dendritic branches was significantly reduced in *EfnB1^Y/−^* samples (88 µm±12) compared to *EfnB1^Y/+^* controls (125 µm±7) (Fig. 6*D*). In addition, we observed varicosities on dendrites from *EfnB1^Y/−^*samples (Fig. 6*B, F*) that are not seen in *EfnB1^Y/+^* controls (Fig. 6*E*). Dendritic spines were then analyzed on the basilar tree of the pyramidal neurons. The number of morphologically distinct dendritic spines on the basilar tree of cortical layer II/III pyramidal neurons was significantly different between *EfnB1^Y/+^* and *EfnB1^Y/−^* samples (Fig. 6*E, F*). Long, thin spines were prevalent on dendritic varicosities of neurons from *EfnB1^Y/−^* mice (Fig. 6*F*). Analysis of spine morphology revealed that neurons in *EfnB1^Y/−^* cortices have a significantly higher number of thin spines and reduced numbers of mushroom or stubby shaped spines compared to neurons in *EfnB1^Y/+^* controls (Fig. 6*G*). Analysis of the total number of spines along a dendrite showed fewer spines throughout the entire length of the basal dendrites of neurons from *EfnB1^Y/−^* samples (Fig. 6*H*).

## Discussion

Here we show that loss of ephrinB1 leads to specific cognitive deficits in adult mice. *EfnB1* deficient mice exhibit defects in non-spatial learning and memory but performed equally to the *EfnB1^Y/+^* controls in all spatial learning tasks. Strikingly, while mutant mice were able to detect spatial differences in the object location task, all subjects showed impairment in the object recognition task. Mutant mice also displayed a significant memory retention deficit in the passive avoidance task and in the fear conditioning paradigm. While ephrinBs were recently implicated in neuropathic pain responses [44], we found no difference in the response of wild type and *EfnB1* mutant mice to the shock in the fear conditioning experiment, indicating that all subjects were able to sense pain in these learning paradigms. These findings demonstrate that the absence of ephrinB1 does not lead to a general cognitive impairment, but rather to a highly selective deficit. To the best of our knowledge, this is a type of cognitive phenotype affecting specifically non-spatial learning and memory has not been reported to date.

Non-spatial learning and memory employs a number of different structures including cortical regions such as the entorhinal and perirhinal cortices, sub-cortical structures such as the amygdala and striatum, and occasionally the hippocampus [45], [46]. Using a number of different learning paradigms with distinctive methodologies, we aimed to identify the most probable brain structure impaired in absence of ephrinB1. For example, the cortex, amygdala and striatum are involved in passive avoidance [46] and fear conditioning [27], [28], [47], [48] and so are potential candidate structures to explain the performance deficit in *EfnB1* mutant mice. However, the striatum and amygdala are not implicated in the object recognition task suggesting that a different structure may be involved in the behavioural deficits observed in our mutant mice. The non-spatial learning tasks are not completely independent of hippocampal functioning as these tests are conducted in a specific context. It is possible, however, to reduce hippocampal involvement by reducing the complexity of the contextual environment [36]. For this reason, we used the object recognition task with the simplest possible context; the passive avoidance task was performed with a very small light chamber in a dark experimental room; and the fear conditioning task employed two different procedures, one in the same context and the second in a different context. As we obtained a deficit in all these procedures, this strongly suggested that the deficit is not context dependant and therefore is independent of hippocampal functioning. Given that the *EfnB1* mutant mice displayed no impairment in either of the spatial learning paradigms employed, which strongly involve the hippocampus, we concluded that the most likely structure implicated in learning and memory deficit in *EfnB1* mutants is the cortex and we thus focused our neuroanatomical analyses on the cortex.

Interestingly, we could not detect ephrinB1 expression in the adult cortex or hippocampus at the time the behaviour studies were performed. While an earlier study has shown widespread diffuse ephrinB1 expression in adult brain in mice [49], we show here, using the *EfnB1* knockout mouse line as a control, that ephrinB1 is not expressed in the adult cortex or hippocampus. Therefore our findings suggest that ephrinB1 does not control learning and memory through direct modulation of synaptic transmission or synaptic plasticity, but instead plays a role in postnatal cortical maturation. Given that ephrinB2 and ephrinB3 have been shown to directly modulate hippocampal synaptic plasticity and learning and memory [50], [51], our findings show that ephrinB1 impacts learning and memory through a mechanism that is distinct from other ephrinBs. Further analyses of *EfnB1^Y/−^* adult brain showed a significant increase in the number of neurons only in the cortex of *EfnB1^Y/−^* mice. The increase in the number of neurons was traced to a postnatal event since neuron numbers were not significantly different between wild type and *EfnB1* mutant mice at birth (P0). How loss of ephrinB1 leads to an increased number of cortical neurons is unknown, yet the timing of appearance of this phenotype is suggestive of a decrease in programmed neuronal cell death that normally takes place in the cortex around P5. A number of studies have shown a potential role for Eph receptors and ephrins in brain region specific apoptosis (reviewed in [52]). Previous work has linked EphA/ephrinA signalling to the control of cell number in the developing brain, via a direct effect on survival of embryonic neural progenitors [53]. While these studies all show a role for Eph/ephrin signaling on apoptosis of neural progenitors, this is the first report to show that a member of the Eph/ephrin family, ephrinB1, is important to control neuron number in the postnatal cerebral cortex. Future work is required to understand the role of ephrinB1 in this apoptotic molecular cascade. Interestingly we noted no difference in cortical thickness from *EfnB1^Y/+^* and *EfnB1^Y/−^* cortices despite changes in cortical neuron numbers. This finding suggests that neuronal packing may be changed in absence of ephrinB1.

In correlation with the increased neuronal numbers, we found aberrant dendritic morphology and arborisation, along with a significant decrease in spine density on pyramidal cortical neurons in *EfnB1^Y/−^* cortices. This reduced branching of the neurons from *EfnB1^Y/−^* mice could explain the maintenance of cortical thickness in *EfnB1^Y/−^* mice despite changes in cortical neuron numbers. Whether reduced branching is a consequence of increased neuron numbers is currently unknown. Neurons from *EfnB1^Y/−^* mice displayed dendritic varicosities, a peculiarity seen in some cases of infantile neurobehavioral failure, in addition to a number of progressive neurodegenerative disorders including Huntington’s disease frontal lobe dementia and Alzheimer’s disease (reviewed in [54]). The cause of the varicosities is not well known, yet varicosities are related to dendritic spine dysgenesis and loss. Closer scrutiny of dendritic spines revealed a significantly higher number of thin spines on neurons in *EfnB1^Y/−^* mutants with reduced mushroom or stubby shaped spines. Spines form a variety of shapes and sizes that correlate with their synaptic strength, motility, and structural plasticity [55]. Evidence shows that thin spines are abundant in the early postnatal brain and undergo refinement to promote dendritic spine maturation and synapse/dendrite stability [2], [54]. Previous studies have shown that stimulation of cultured neurons with soluble recombinant ephrinB1 protein induces dendritic spine formation and maturation [20], [56]. These findings suggest a direct role of ephrinB1 in spine maturation and suggest that the abundance of thin spines in *EfnB1^Y/−^*cortices could be a manifestation for the privation of postnatal spine maturation in the absence of ephrinB1. An increase in thin spines has been associated with several hereditary forms of intellectual disability, and is often observed for many genes that underlie X-linked intellectual disability [57]. It is thus possible that the defective architectural structuring of synaptic connections in *EfnB1^Y/−^*cortical pyramidal neurons underlies cognitive impairment of these mutants. Alternatively, changes in the number of CR+ cells have been reported in various disorders [58], and we have observed an alteration of the CR+ interneuron subtype in the somatosensory cortex of *EfnB1* mutant mice. To date the functional properties of CR+ interneurons have been primarily studied in the hippocampus where they are responsible in synchronizing dendritic inhibitory cells [59] to regulate input information into principal neurons [60]. Why CR+ interneurons were increased specifically in the S2 cortical region of *EfnB1^Y/−^*mutants and whether this increase contributes to the behavioural impairment observed in these mice remains unknown. Overall our findings suggest that in the absence of ephrinB1, postnatal neuronal cell death does not occur normally, thus leading to imbalance between neuronal subtypes and to improper maturation of dendritic arbors.

Mutations in *EfnB1* are associated with a human syndrome called CranioFrontoNasal Syndrome (CFNS) [16], [17]. CFNS is a rare X-linked disorder that shows paradoxically greater severity of craniofacial abnormalities in heterozygous females than in hemizygous males. This is due to the fact that these abnormalities are exacerbated by cellular mosaicism due to random X-inactivation in female patients. This peculiar feature of CFNS is also observed in mice, since heterozygous females present more penetrant and generally stronger -yet similar- phenotypes than hemizygous males [61], [62]. To date there is only sporadic information on the cognitive capacity of CFNS male or female patients; however, our study suggests that this syndrome may share a number of characteristic features with other neurodevelopmental disorders. Interestingly, cortical anomalies observed in *EfnB1^Y/−^* mutants also partially resemble those seen in genetic models for Fragile-X Syndrome, which exhibit a striking inhibitory/excitatory synapse imbalance and disrupted synaptic contacts [63]. Altogether, our findings demonstrate that ephrinB1 is involved in cortical maturation and in non-spatial learning and memory.
